# Exosomes in Intestinal Inflammation

**DOI:** 10.3389/fphar.2021.658505

**Published:** 2021-06-09

**Authors:** Kanchana K. Ayyar, Alan C. Moss

**Affiliations:** Division of Gastroenterology, Department of Medicine, Boston Medical Center, Boston, MA, United States

**Keywords:** IBD, colitis, extracellular vesicles, exosomes, inflammation

## Abstract

Exosomes are 30–150 nm sized vesicles released by a variety of cells, and are found in most physiological compartments (feces, blood, urine, saliva, breast milk). They can contain different cargo, including nucleic acids, proteins and lipids. In Inflammatory Bowel Disease (IBD), a distinct exosome profile can be detected in blood and fecal samples. In addition, circulating exosomes can carry targets on their surface for monoclonal antibodies used as IBD therapy. This review aims to understand the exosome profile in humans and other mammals, the cargo contained in them, the effect of exosomes on the gut, and the application of exosomes in IBD therapy.

## Introduction

Exosomes are biological nanovesicles (30–150 nm) that are made by most cell types (Gross et al., 2012). They fuse with the plasma membrane of the cell and are released outside, they can be detected in almost all biological fluids (Edgar, 2016). The exosomal membrane is made of lipids and proteins (Mathivanan et al., 2010). The cargo contained within the exosomes can be proteins, nucleic acids and lipids which may or may not be similar to those of the parent cell (Valadi et al., 2007). Neighboring and/or distant cells can benefit from the cargo of circulating exosomes. They function as signaling molecules and can bring about regulation. The knowledge of their autocrine, paracrine, juxtacrine, and endocrine regulation has aroused the curiosity of many researchers. Studies indicate that exosomes play a role in extracellular matrix remodeling and signal transmission (Beshbishy et al., 2020). They also play vital roles in many disease conditions like cancer, neurodegenerative disorders, cardiovascular diseases, diabetes and others like Inflammatory Bowel Disease (IBD) by regulating immunity, inflammation, and tissue homeostasis.

Inflammatory Bowel Disease (IBD) has been widely thought to be a western disease; however, the global prevalence of IBD has been increasing since 2000. One in 200 individuals in Western countries suffers from IBD (Ng et al., 2017). Crohn’s disease (CD) and ulcerative colitis (UC) are the two distinct disorders of IBD. CD and UC differ in the symptoms, complications, part of the gastrointestinal (GI) tract they affect, pathophysiology, disease course and management. UC affects the colon and is characterized by continuous lesions and superficial inflammation, which can lead to erosions, ulcers and bloody diarrhea. CD is progressive and destructive—many patients have systemic, extra intestinal manifestations, which strongly affects their quality of life due to risk of hospitalization, complications and surgery (Torres et al., 2017).

The pathogenesis of IBD is still ambiguous, the process is linked to genetic, environmental, gut microbiota and immune response factors (Ocansey et al., 2019). Some individuals with IBD have a genetic predisposition due to which there is a failure in maintaining intestinal homeostasis (Console et al., 2019). Intestinal Epithelial Cells (IECs) are constantly exposed to numerous bacteria and dietary-derived antigens. In order to defend itself, the human body needs to mount a robust immune response against invading pathogens, however if this response is prolonged it can be deleterious to the host leading to acute and chronic inflammatory disorder. Thus, the cells of the body employ intricate signaling network to control inflammation (Ayyar and Reddy, 2018). This system includes many small extra cellular components such as extra-cellular vesicles (EV), non-coding RNAs (ncRNA) like miRNA (microRNA), snRNA (small nucleolar RNA), siRNA (small interfering RNA), lncRNA (long non-coding RNA), and others (Ayyar et al., 2014). The immune system of the intestinal mucosa employs the use of such extra-cellular factors such as exosomes to maintain a balance between exhibiting tolerance toward normal microbiota and food proteins and eliciting an immune response toward enteric pathogens. Exosomes regulate cells of the immune system, gut microbes and factors maintaining the gut barrier within the IBD microenvironment. This helps repair damage and restore intestinal mucosal functions. IBD therapy is directed at improving immune regulation and easing intestinal mucosal inflammation. Exosome based therapy can help achieve these goals. In this article, we review the contents of exosomes and their effects in IBD; focusing on their effect on immune cells and gut microbiota. We also examine the potential applications of exosomes and exosomal cargo as biomarkers of IBD and in IBD therapy.

## Exosomes

Exosomes, prostasomes, ectosomes, microvesicles, microparticles, tolerosomes, and nanovesicles are collectively referred as EVs. Currently, exosomes cannot be easily separated from other EVs (Beshbishy et al., 2020). Thus, exosomes separated from biological fluids and cell culture supernatants that are used for research are regarded as EVs, as stated in the guidelines developed by the International Society of Extracellular Vesicles (ISEV) (Théry et al., 2018). Existence of EVs was documented for the first time in 1946 (Chargaff and West, 1946). By 1977, the release of membrane fragments was considered a ubiquitous feature of viable cells. Till 1980, they were commonly considered as cellular debris (P. Wolf, 1967). The discovery of exosomes occurred in 1983. Pan and Johnstone reported that during maturation of reticulocytes in sheep, small vesicles were engaged in the release of transferrin receptors into the extracellular milieu (Pan and Johnstone, 1983). These vesicles were termed exosomes in 1989 (Johnstone et al., 1989). Subsequent studies demonstrated that exosomes originate from endosomal vesicles.

### Biogenesis of Exosomes

Exosomes have a lipid bilayer membrane and are incapable of replicating. The process by which exosomes are synthesized differs from other EVs. These vesicles are forged from a novel multi-step “exosome biogenesis” pathway. The plasma membrane of the cell buds inwards to form membrane enclosed compartments called early endosomes (Pant et al., 2012). Early endosomes mature into late endosomes, these are also called multivesicular bodies (MVB). MVBs acquire intraluminal vesicles (ILVs) in their lumen. Inward budding of early endosomal membrane results in generation of ILVs. ILVs contain biologically active molecules like lipids (Subra et al., 2010), proteins (André et al., 2004) and nucleic acids (Valadi et al., 2007). These components may be selectively enriched by the endosomal sorting complexes required for transport (ESCRTs) (Tamai et al., 2010). The process may also be orchestrated by ESCRT-independent mechanisms which involve tetraspanins or lipids (Colombo et al., 2013). Depending on the molecules used, the mechanism is considered ESCRT dependent or ESCRT independent (Stuffers et al., 2009). The resultant MVBs can have two fates: survive as exosomes or perish. Fusion of MVBs with plasma membrane leads to their release in extracellular space as exosomes (Gruenberg and Goot, 2006). Alternatively, MVBs may fuse with lysosomes which leads to degradation of their content (Palmulli and Van Niel, 2018). Exosomes are released by exocytosis pathway in the course of cellular crosstalk or during receptor removal mechanisms. Growth factor receptors on the plasma membrane play a pivotal role in the exocytosis pathway (Stoorvogel et al., 2002). It is not yet fully understood as to how exosomes are released. Even though considerable progress has been made in this field, substantial gap exists in our knowledge of cargo sorting mechanisms in exosomes and other vesicular bodies.

### Characterization of Exosomes

A few minimal requirements have been made mandatory by ISEV to establish the existence of exosome in a study. Experimental methods like electron microscopy, concentration monitoring techniques, and western blotting are needed to identify the presence of exosomes (Théry et al., 2018). Also, physical properties like particle size are calculated during isolation via ultracentrifugation, density gradient separation, and polymer-based precipitation methods. (Chang et al., 2020). Under TEM (Transmission Electron Microscopy), exosomes resemble cup-shaped lipoidal vesicles (Théry et al., 2009). Most exosomes bear tetraspanin proteins and this superfamily of proteins was considered to be a definitive marker of exosomes. Recent research indicates that micro-vesicles (MVs) also bear CD63, CD9, and CD81 tetraspanin proteins (Simons and Raposo, 2009). Many studies on exosomes have shown Alix and TSG101 (Tumor Susceptibility Gene 101 protein) and heat shock proteins HSC70 and HSP90 to be associated with these vesicles (Maas et al., 2017). The main difference between endosomes and MVs is that MVs are derived from outward blebbing of the plasma membrane (Agrahari et al., 2019).

### Trafficking of Exosomes

The cell to cell communication by exosomes can occur via three mechanisms. The first mechanism involves receptor-ligand interaction. Signaling receptors of target cell interact directly with exosomal transmembrane proteins (Munich et al., 2012). In the second mechanism, the plasma membrane of target cell fuses with exosome, the contents of the exosome is directly delivered into the cytosol (Mulcahy et al., 2014). In the third mechanism, the exosomes get internalized by target cells. The internalized exosomes may either merge with endosomes, undergo transcytosis and be released for uptake by neighboring cells or the exosomes mature into lysosome in the target cells and are degraded (Tian et al., 2013). Some of the factors that influence internalization of exosomes have been studied. Exosomal lipid rafts and annexins are essential for exosomal internalization (Koumangoye et al., 2011). This is not to say that all factors responsible for internalization of exosomes are present only on exosomes. The proteins mediating internalization of exosomes are present on both target cells and exosomes (Escrevente et al., 2011). Apart from proteins, alteration of cholesterol content leading to disruption of lipid rafts also impairs exosomal internalization (Gonda et al., 2019). We still lack knowledge of how a mechanism is preferred over another by the exosome and/or the cell; is it condition, cell type or exosome specific?

### Exosomal Cargo

Cell-to-cell communication is essential and exosomes help in facilitating this cellular crosstalk (Carrière et al., 2016). The lumen of the exosome contains the cargo-which could be lipids, proteins, nucleic acid like mRNAs, microRNAs, and other non-coding RNAs (ncRNA) (Sato-Kuwabara et al., 2015). The cargo can be same or different from that of the parent cell. Exosomes contain proteins primarily present in endosomes, plasma membrane and cytosol. They rarely enclose factors originating from the nucleus, mitochondria, endoplasmic reticulum or golgi. Also, formation and secretion of exosomes is a cost intensive process for the cell requiring enzymes and ATP (adenosine triphosphate) (J. Zhang et al., 2015). We believe there is mechanism in the cell for actively selecting exosomes and their cargos.

#### Proteins

Exosomal research data from different sources reveal that they share common exosomal constituents. As of 2016, 41,860 exosomal proteins, >7,540 exosomal RNA, and 1,116 exosomal lipid molecules have been cataloged from more than 286 exosome-based studies defined as per ISEV guidelines (Keerthikumar et al., 2016). Exosomes have two different kinds of protein content. The first kind depends on the cell and tissue from which it originates, while the other kind of protein constitutively occurs in exosomes and can be used as exosomal markers (Console et al., 2019). TSG101, Charged Multivesicular Body Protein 2a (CHMP2A), Ras-related protein Rab-11B (RAB11B), CD9, CD63, and CD81 are currently used as exosomal protein markers (Gross et al., 2012). Other common proteins documented in exosomes include adhesion molecules, heat shock proteins (such as HSC73, HSC90), annexins I, II, V, and VI, cytoskeletal proteins (synenin, actin, moesin, albumin) and GTPases (Samanta et al., 2018). Interestingly, some cytoskeletal proteins, glycolytic enzymes, and argonaute 1–4 (required for miRNA stability) are not detected in exosomes (Jeppesen et al., 2019).

#### Lipids

The current knowledge regarding the lipid content of exosomes is limited. Exosomes may contain cholesterol, sphingomyelin, ceramide, and phosphatidylserine. Cholesterol was found in the membrane of internal vesicles of MVB (Möbius et al., 2003). This is relevant as many membrane transporters are sensitive to cholesterol (Dickens et al., 2017). The structure of exosomal membrane is analogus to that of the plasma membrane; it also contains detergent resistant subdomains (Colombo et al., 2014).

#### Nucleic Acids

The other cargo contained within exosomes is nucleic acids (DNA, mRNA, miRNA, lncRNA, and ncRNA). The delivery of nucleic acids to cells can alter expression of genes and regulate the immune status in neighboring tissue. miRNAs can modulate immune responses by production and release of cytokines and chemokines resulting in a feedback regulation of immune homeostasis (Ayyar and Reddy, 2017). The nucleic acid content within exosomes differ. Identifying them will help us understand the role they may play in many processes. As exosomes are produced by almost all cells, they can be harvested from physiological fluids and their contents may indicate health of the tissues (healthy vs. diseased). They have the potential to be used as biomarkers: a diagnostic tool. A recent study suggests that the DNA found in small EVs are of extracellular origin that are released via an autophagy and multivesicular-endosome-dependent but exosome independent mechanism (Jeppesen et al., 2019). The source of different EV components and their functions is listed in Table 1.

**TABLE 1:** Source and function of different extracellular vesicle components in IBD.

Exosomal component	Source	Role	References
A33 antigen	Intestinal epithelial cells	Cell-cell recognition and signaling	Van Niel et al. (2001)
Annexin A1	Intestinal epithelial cells	Promotes wound healing	Leoni et al. (2015)
CCL20 and prostaglandin E2	Intestinal epithelial cells	Recruit Th17 cells through MyD88 mediated pathway	Deng et al. (2015)
CD63 and EpCAM	Intestinal epithelial cells	Induces DC apoptosis	Kojima et al. (2018)
		Suppresses DC maturation inhibits antigen presentation by DCs in rats	
GELNs lipids	Grapes	Induces Lgr5+ stem cells	Ju et al. (2013)
		Enhances *in vivo* targeting of intestinal stem cells	
		Remodels and protects intestinal tissue against DSS-induced colitis	
Integrin αvβ6	Intestinal epithelial cells	Promotes production of active TGF-β in DCs and Tregs	Chen et al. (2011)
Metallothionein-2	Bone marrow-derived MSCs	Maintenance of intestinal barrier integrity	(Liu et al. (2019)
		Polarization of M2b macrophages	
		Induction of IL-10 from macrophages	
MHC I and II	Intestinal epithelial cells	Initiating immune response	Van Niel et al. (2001)
miR-223	Mice colonic epithelial cells	Modulates communication between IL-23 signal pathway and claudin-8 in IBD development	Wang et al. (2016)
		Modulates intestinal barrier integrity	
miR-23a and miR-155	Neutrophils	Induces replication fork collapse	Butin-Israeli et al. (2019)
		Inhibits homologous recombination	
		Induces accumulation of double strand breaks	
miR-34c and PlncRNA1	Intestinal epithelial cell line Caco-2	Modulates ZO-1, MAZ, and occludin expression	Chen et al. (2017)
		Modulates intestinal barrier integrity	
miR-4334, miR-219 and miR-338	Porcine milk	Prevents LPS-induced intestinal inflammation, apoptosis and damage via inhibiting TLR4/NF-κB and p53 pathways	Xie et al. (2019)
Mdo-miR7267–3p	Ginger	Shapes gut microbiota	Teng et al. (2019)
		Improves barrier function	
		Ameliorates colitis via IL-22-dependent mechanisms	
Myeloperoxidase (MPO)	Intestinal epithelial cells	Contributes to oxidative stress against microbes	Zhang et al. (2018)
Myeloperoxidase (MPO)	Neutrophils	Damages intestinal barrier by production of oxidative radicals, inhibiting wound closure and healing	Slater et al. (2017)
NEAT1	Mouse intestinal mucosa and serum	Down-regulation of NEAT1	Liu et al. (2018)
		Suppresses inflammatory response by modulating intestinal epithelial barrier and via exosome-mediated polarization of macrophages in IBD	
PSMA7	Oral mucosal cells	Responsible for degradation of proteins	Zheng et al. (2017)
		Controls autoimmune disorders and immune tolerance	
Sphingosine-1-phosphate	Intestinal epithelial cells	Promotes tumorigenesis	(Patmanathan et al. (2017)
Tetraspanin 14–3-3 protein, enolase and heat shock proteins	Hookworm	Protection against colitis by significantly suppressing	(Eichenberger et al. (2018)
		IFNγ, IL-6,IL-1β, and IL-17a and upregulating anti-inflammatory cytokine IL-10	
TGF-β	Intestinal epithelial cells	Inhibit CD4^+^ T cell proliferation	Jiang et al. (2016)

### Exosomes in Inflammatory Bowel Disease

The role of exosomes in IBD has garnered interest among researchers. Exosomal proteins, RNAs and lipids regulate factors of IBD like immune cells, gut microbiota and the mucosal barrier (Chang et al., 2020). Many studies have used exosomes from a variety of starting materials to study the process of IBD alleviation and related factors of interest. Translational studies are being conducted to tap into the possible use of exosomes as diagnostic markers and drug delivery systems, and also on the utility of modified exosomes in IBD therapy (Ocansey et al., 2020). Many of the studies cited in this review do not differentiate between the different types of EVs like exosomes, endosomes and MVBs, hence for the sake of convenience we have used the general term EVs while describing study findings.

### Exosomal Profile in Intestinal Mucosa

Epithelial cells, Paneth cells, macrophages, and lymphocytes are cells in the intestinal tract that are directly exposed to digested food, microbes, and foreign antigens making immune homeostasis a challenging and complex process (Peterson and Artis, 2014). While maintaining immune homeostasis, the intestinal barrier strikes a perfect balance by clearing pathogenic bacteria and maintaining immune tolerance with the commensals. Evidence indicates that dysregulation of intestinal homeostasis leads to IBD pathogenesis. Intestinal homeostasis depends on effective crosstalk between extracellular factors, EVs and the host’s intestinal immune defense (comprised of the mucus membrane, intestinal epithelial cells (IECs) and the immune cells) (Avila-Calderón et al., 2015). EVs act not only as communicator between cells but also between cells and organisms (Chang et al., 2020). This is important as the mammalian intestine encounters 10 trillion (10^13^) microbes (10X the number of total cells in a mammalian body) (Sartor, 2008). EVs from micro-organisms and the host body communicate and help maintain a peaceful coexistence, maintaining intestinal immune homeostasis which is a major determinant of health (Sommer et al., 2017). Physiological fluids also contain EVs which affect the intestinal microbiota.

#### Exosomes From Intestinal Epithelial Cells

IECs play a major role in immune modulation in the gut that even though they are not professional antigen presenting cells (APC), they bear major histocompatibility complex (MHC) I, II, and HLA-DM (Lin et al., 2005). The knowledge of IEC releasing EVs has been known for a while now (Van Niel et al., 2001). IEC EVs like parent cells, contain immunomodulatory molecules. They also express MHC I and II, expression of which is elevated during inflammation as compared to basal condition (Van Niel et al., 2001). EVs released from these cells can be released apically or basolaterally. They carry A33 antigen, whose expression is confined to the intestinal epithelium (Van Niel et al., 2001). A33 is now considered a marker for IEC exosomes (Van Niel et al., 2003; Lin et al., 2005; Mallegol et al., 2007).

**FIGURE 1:**
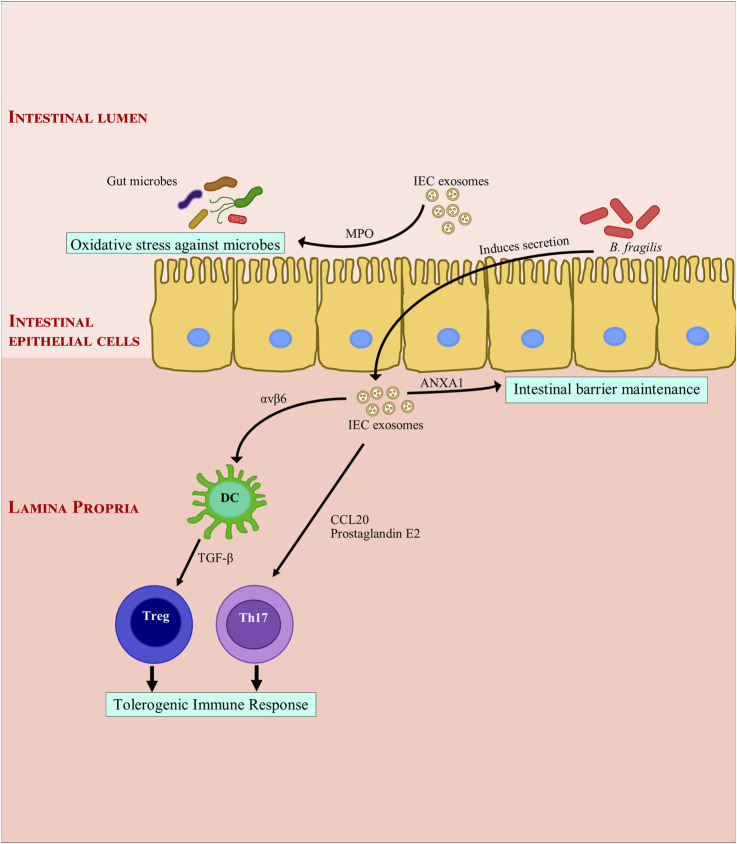
Modulation of intestinal inflammation by intestinal epithelial cell derived exosomes. Intestinal epithelial cell derived exosomes help maintain gut immune homeostasis through secretion of Annexin A1. They induce a tolerogenic immune response by secretion of integrin, cytokines and chemokines. They also protect the gut immune barrier from bacterial invasion by secreting myeloperoxidase which creates oxidative stress against bacteria. ANXA1, Annexin A1; αvβ6, Integrin αvβ6; *B. fragilis*, *Bacteroides fragilis*; CCL20, C-C Motif Chemokine Ligand 20; DC, dendritic cell; IEC, Intestinal epithelial cells; MPO: myeloperoxidase; Th17, TGF-β, transforming growth factor β; T helper 17; Treg, regulatory T cell.

IEC EVs are known to interact with Dendritic cells (DC) (Mallegol et al., 2007). They stimulate DC, Treg and macrophage maturation with tolerogenic properties through immunoregulatory signals (Jeffrey et al., 2018). Also, tolerogenic DCs are essential for maintaining intestinal homeostasis (Peterson and Artis, 2014). The cytokine, TGF-β is produced in tolerogenic DCs and Tregs in its latent form as LTGF-β. IEC EVs carrying integrin αvβ6 are internalized by intestinal tolerogenic DCs, wherein they help activate TGF-β, induce Treg cells and initiate tolerogenic responses in the gut (Chen et al., 2011). CD4^+^ T cell proliferation is inhibited by TGF-β containing IEC EVs (Jiang et al., 2016). IEC EVs have a wide spectrum immuno modulatory effect. EVs expressing CD63 and EpCAM (Epithelial cell adhesion molecule) suppress DC maturation and inhibit antigen presentation by DCs and also induce DC apoptosis in post-trauma immune dysfunction in rats (Kojima et al., 2018). EpCAM plays a unique role in immuno-modulation by facilitating a physical interaction between intraepithelial lymphocytes and IECs (Jiang et al., 2016). Annexin 1 (ANXA1) is crucial for maintaining the intestinal barrier during inflammatory response; it is found in exosomes released from IECs (Leoni et al., 2015). These ANXA 1 containing EVs help in resolving inflammation in murine colitis, their number increases during wound healing (Leoni et al., 2013; Perretti and D’Acquisto, 2009). These EVs bind to formyl peptide receptors (FPRs) and may be employed to activate wound repair in epithelial cells (Leoni et al., 2015). In IBD patients, mucosal-luminal interface EVs express MPO (myeloperoxidase). MPO is a defense protein which creates oxidative stress against microbes in the intestines (X. Zhang et al., 2018). At the same time, intestinal fluid EVs from IBD patients have a pro-inflammatory effect on IECs *in-vitro* (Mitsuhashi et al., 2017). This disparity can be attributed to the source of EVs. EVs secreted by *Bacteroides fragilis* induce secretion of host mucosal EVs containing sphingosine-1-phosphate, CCL20, and prostaglandin E2 (Deng et al., 2015). Th17 cells are sequestered by Prostaglandin E2 and CCL20 via MyD88-mediated pathway (Deng et al., 2015), while sphingosine-1-phosphate plays a role in tumorigenesis (Kunkel et al., 2013; Hait and Maiti, 2017; Patmanathan et al., 2017). Conversely, it is possible that CCL20 and other pro-inflammatory cytokines are inhibited by EVs derived from healthy intestinal mucosa (Deng et al., 2015). Figure 1 summarizes exosomal effects of IEC derived exosomes on intestinal barrier functions and immunity.

#### Exosomes From Immune Cells

The mucosal barrier maintains immune homeostasis in the gut by spatially separating the gut microbiota from the host immune system. In IBD, there is dysfunction of the barrier, immune dysregulation and dysbiosis. Both the innate and the adaptive immune system signals lead to IBD pathogenesis. The innate immune response is quicker; it involves phagocytosis, antigen presentation and provides stimulus for initiation of the adaptive immune response. The process involves macrophages, DCs, neutrophils, and monocytes (Ayyar et al., 2014). Many APCs have been reported to secrete exosomes, these exosomes may also bear MHC molecules (Morelli et al., 2004).

DCs are professional APCs of the immune system and can initiate an immune response upon sensing antigens. Based on the stage and maturation of parent DCs, DC derived exosomes may have immune stimulatory/suppressive effects (Lindenbergh and Stoorvogel, 2018; H.; Zhang et al., 2019). EVs from DCs can inhibit T-cell proliferation, this could play a key role in modulating inflammation in IBD (Kim et al., 2007; Tkach et al., 2017). Mature DC-derived exosomes that contain tumor antigens potentially could induce anti-tumor immunity in *in-vitro* trials, while immature exosomes could induce peripheral tolerance by T-cell immunosuppression (Yang et al., 2010). TGF-β1-modified bone marrow derived DCs (BMDC) produce exosomes that induce CD4^+^Foxp3^+^Tregs and attenuate Th17 population in lymphocytes from mesenteric lymph nodes of inflammatory site in DSS induced mice (Cai et al., 2012). Similarly, IL-10 treated DC exosomes inhibit 2,4,6-trinitrobenzenesulfonic acid (TNBS) induced colitis in rats by stimulating CD4^+^CD25^+^Tregs (Yang et al., 2010). In a similar study, EVs from *Schistosoma japonicum-*soluble antigen-treated DCs provided protection during acute IBD development (Tkach et al., 2017; L. Wang et al., 2017). Immune tolerance can be achieved through regulation of activated T cells, by harnessing the immunosuppressive activity of exosomes. APC derived exosomes can activate CD4^+^ and/or CD8^+^ T lymphocytes, their antigen presentation capacity relies on DC, indicating that APCs take up DC derived exosomes to promote T cell activation. Exosomes may quickly transfer immune information via APCs (Van Niel et al., 2003).

**FIGURE 2:**
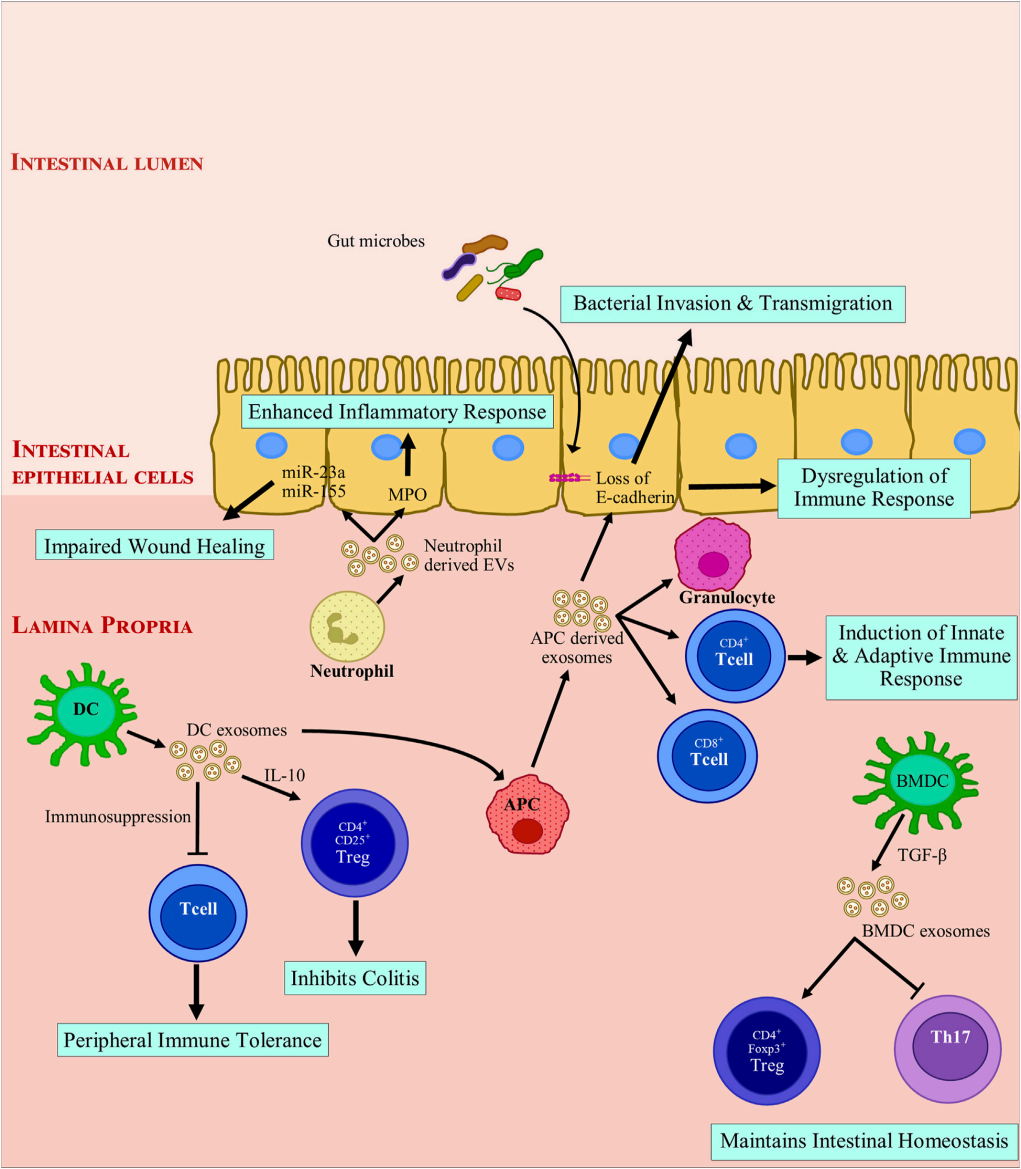
Modulation of intestinal inflammation by immune cell derived extracellular vesicles. Exosomes derived from immune cells promote anti-inflammatory responses by inducing immune tolerance and triggering regulatory T cells (Treg) activation while inhibiting T helper cells. Exosome-treated immune cells further express exosomes that encourage anti-inflammatory responses. Mature APC derived exosomes promote loss of E-cadherin, which leads to breach of barrier integrity and facilitates bacterial invasion and transmigration. These exosomes also recruit immune cells and drive a pro-inflammatory immune response. Neutrophil derived exosomes secrete myeloperoxidase and miRNAs which are taken up by intestinal epithelial cells. miR-23a and miR-155 can introduce double strand breaks and impair wound healing in degenerated colonic epithelium. In summary, depending on the parent cells, exosomes derived from immune cells can drive toward a pro-inflammatory or an anti-inflammatory response. APC, antigen presenting cell; BMDC, Bone marrow derived dendritic cell; CD, cluster of differentiation; DC, dendritic cell; EV, extracellular vesicles; FOXP3, Forkhead box protein 3; IL-10, Interleukin 10; miR, microRNA; MPO: myeloperoxidase; TGF-β, transforming growth factor β; Th17, T helper 17; Treg, regulatory T cell.

IBD progression is affected in myriad ways by other immune cell derived EVs. Neutrophil infiltration occurs in IBD and is accompanied by liberating MPO into the extracellular space. MPO results in production of oxidative radicals which damage the gut barrier. It was found that MPO was delivered to IEC via EVs and inflammatory response was enhanced, inhibiting wound closure and healing (Slater et al., 2017). EVs also contain miRNAs as cargo; miRNAs are excellent regulators of immune response (Ayyar et al., 2014). EVs containing proinflammatory miRNAs can promote doublestrand breaks (DSBs) in affected colonic epithelia (Butin-Israeli et al., 2019). Neutrophil derived EVs can contain miR-23a and miR-155. These EVs promote lamin B1- dependent replication fork collapse and also suppress homologous recombination (HR) by repressing RAD51 (Bui and Sumagin, 2019; Butin-Israeli et al., 2019). During transepithelial migration of granulocytes, granulocyte EVs accumulate on IECs. This promotes the recruitment of granulocytes accompanied by a loss of epithelial cadherins (Butin-Israeli et al., 2016). On the other hand, granulocytic myeloid-derived suppressor cells-derived EVs diminish number of Th1 cells and a resultant surge is observed in Treg population in colitis induced mice (Y. Wang et al., 2016). WNT signaling is essential for intestinal epithelium and homeostasis (Kuhnert et al., 2004; Gregorieff et al., 2005). Intestinal stem cells can be rescued by macrophage-derived EVs. They can also improve enterocyte survival post radiation via modulation of WNT signaling pathway (Saha et al., 2016). Figure 2 shows the general modulatory features of immune-cell-derived exosomes on the immune system.

### Exosomal Profile in Animals

Many studies have been conducted in rats and mice to study the effects exosomes in IBD. A radical difference was observed in the proportion of gut-bacteria derived EVs in feces of DSS (dextran sulfate sodium) induced colitis mice in comparison with those from healthy mice (Kang et al., 2013). The fecal EVs can be IEC-derived, microbiome-derived or may come from the diet. When healthy exosomes from mice were transferred to IBD-induced mice, it was observed that the disease severity markedly reduced in recipient mice (Jiang et al., 2016). This shows that exosomes have therapeutic potential. Another study showed that the protein load of exosomes in healthy and acute-colitis induced mice was different. A total of 56 acute phase proteins and immunoglobulins were found to be differentially expressed between the two groups (Wong et al., 2016). *Lactobacillus* and Bifidobacterium are known probiotics which are administered to IBD patients. DCs and EV isolated from TLR2 (toll-like-receptor 2) knockout mice and healthy mice were co-cultured with these bacteria. It was observed that TLR2/6 activity in DCs was reduced significantly (van Bergenhenegouwen et al., 2014). The effect was reversed upon EV depletion suggesting an immunosuppressive role of these EVs. Fecal OMVs (outer membrane vesicles) from colitis induced rats (DSS) reduced the expression of enzyme UDP-glucuronosyltransferase 1A1 (UGT1A1) in human Caco-2 cells while OMVs from healthy rats upregulated the enzyme expression (X. J. Gao et al., 2018). UGT1A1 helps in maintaining the Intestinal epithelial barrier. This study shows that rat OMVs can modulate mucosal immunity. EVs isolated from mesenteric lymph modulated the immune response through DC suppression in post-trauma immune dysfunction in rats (Kojima et al., 2018). When exosomes are introduced in naïve rats from IBD induced rats, antigen specific tolerance is detected; these exosomes were briefly called tolerosomes (Karlsson et al., 2001).

### Exosomal Profile in Blood

Diseases like Cancer, inflammatory diseases and infections result in change in exosome composition of patients as compared to healthy controls. These changes can be observed in biological fluids like blood and blood components, saliva, stool, urine. These fluids represent an excellent tool for identification of biomarkers (Barile and Vassalli, 2017). Unfortunately, there are not many reports of EVs or exosomes in blood/serum. In 2015, Leoni et al. looked at serum exosome levels in IBD patients. They found that exosomes released from IECs, entered circulation and expressed ANXA 1, which is crucial for maintaining the intestinal barrier during inflammatory response. Serum of IBD patients contain good number of such vesicles (Leoni et al., 2015). Additional research on the serum exosomal content in IBD patients will help better our understanding.

### Role of Dietary EVs in Inflammatory Bowel Disease

The source of exosome existing in human body could be produced by human cells such as immune cells, IECs, tumor cells or by plant cells which are introduced through dietary food. As almost all biological cells produce exosomes; many of the edible foods that we consume contain exosomes.

#### Plant Derived Exosomes

Some studies have been conducted on exosomes derived from edible plants. *Curcuma longa* and grapes contain exosomes that helps in alleviating IBD. *Curcuma longa* is a medical herb, the exosomes derived from this plant may inactivate NF-κB to alleviate colitis and improve intestinal wound repair (H. Zhang et al., 2019). Exosome-like nanoparticles (ELN) from grape juice play a protective role in DSS-induced colitis mice when administered via the oral route (Ju et al., 2013). ELNs from ginger rhizome strongly inhibit NLRP3 (nucleotide-binding domain and leucine-rich repeat-containing family, pyrin domain-containing 3) inflammasome activation. Events occurring downstream of inflammasome activation like secretion of cytokines IL-1β and IL-18, auto cleavage of caspase-1 and pyroptotic cell death can also be impeded by these ELNs (Chen et al., 2019). Broccoli derived ELNs inhibit induced colitis in mice. These ELNs help in the activation of AMPK (AMP [adenosine monophosphate]-activated protein kinase) in DCs, which promotes DC tolerance and prevents DC activation, leading to inhibition of DSS induced colitis in mice (Deng et al., 2017).

#### Milk Derived Exosomes

**FIGURE 3:**
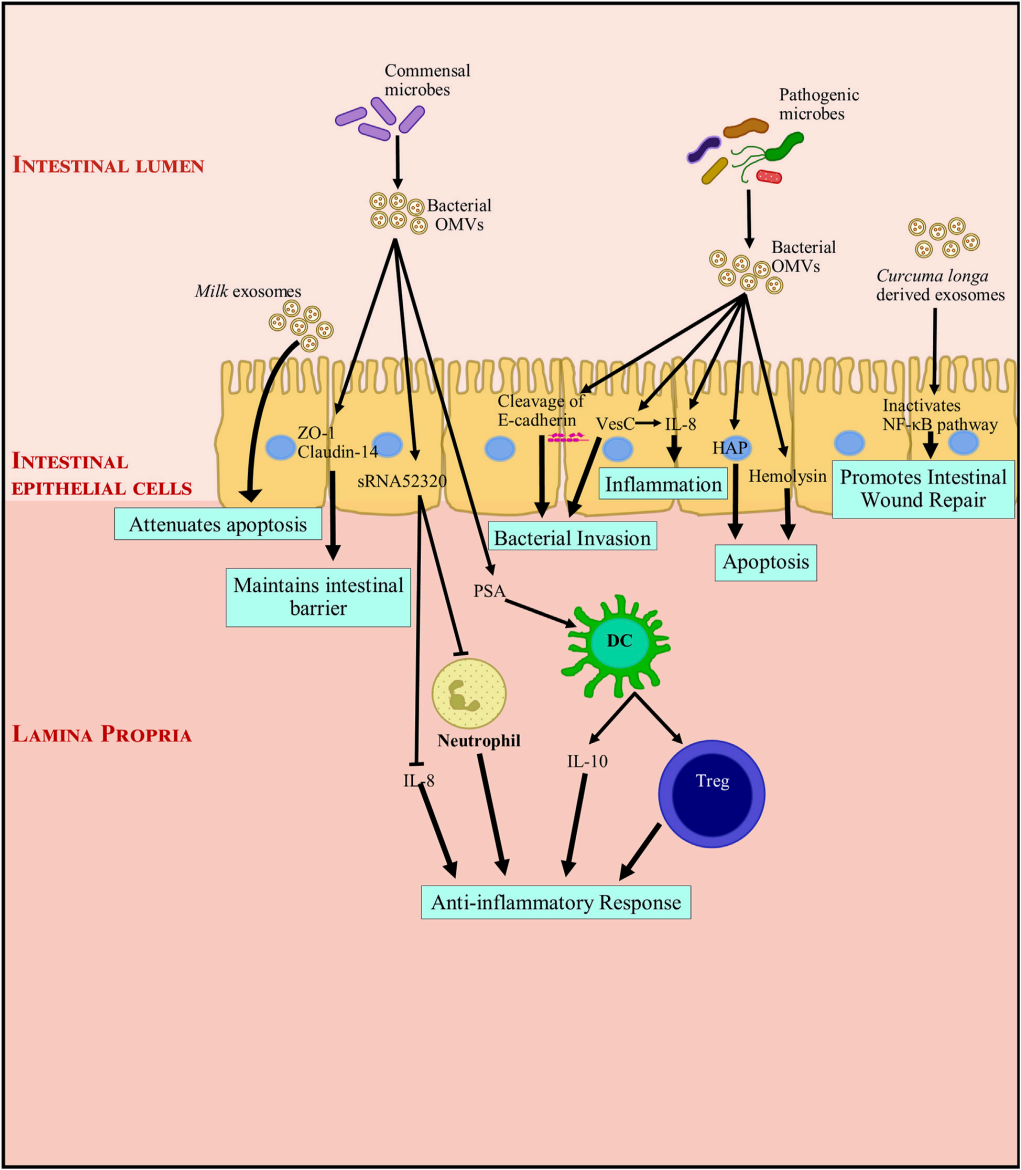
Modulation of intestinal inflammation by microbiome derived outer membrane vesicles and dietary exosomes. Exosomes and OMVs (outer membrane vesicles) through their functional components, directly or indirectly interact with gut intestinal epithelial cells (IECs). Dietary exosomes attenuate apoptosis and promote intestinal wound repair. OMVs from commensal bacteria induce the proliferation of IECs and development of the intestinal tract, and indirectly enhance barrier functions by inhibiting components of the inflammatory environment that negatively impact tight junction molecules and IECs. They also interact with immune cells and promote an anti-inflammatory immune response. On the other hand, pathogenic bacteria, though their OMVs can induce inflammation and apoptosis. They can also cause a breach in the epithelial barrier by cleaving E-cadherin. This facilitates bacterial invasion and transmigration into the intestinal tissue. DC, dendritic cell; HAP, hemagglutinin protease; IL, Interleukin; NF-κB, nuclear factor kappa light chain enhancer of activated B cells; OMV, outer membrane vesicle, PSA: capsular polysaccharide; sRNA52320, shortRNA 52320; Treg, regulatory T cell; VesC, calcium-dependent trypsin-like serine protease; ZO-1, Zonula occludens 1.

Breast milk not only provides nutrition but also helps shape the neonate gut immune system (Turfkruyer and Verhasselt, 2015). Breast milk has immunoglobulin which help in responding precisely to encountered antigens (Walker and Iyengar, 2015; Doare et al., 2018). Recent studies have shown EVs contained in milk can affect intestinal homeostasis (Admyre et al., 2007). Bioactive milk exosomes are known to carry miRNAs, mRNAs, lncRNAs, TGF-β and a variety of other proteins and lipids (Reinhardt et al., 2012; Reinhardt et al., 2013; Pieters et al., 2015; Tomé-Carneiro et al., 2018). Exosomes are resistant to the harsh conditions in the gastrointestinal tract (Benmoussa et al., 2016; Liao et al., 2017; Kahn et al., 2018) but are actively captured by intestinal epithelial cells (T. Wolf et al., 2015). It has been observed that a considerable portion of milk exosomes (from consumed milk) enter circulation in rodents and humans (Baier et al., 2014; Manca et al., 2018; Betker et al., 2019). The fate of unabsorbed exosomes is not yet known. There is strong evidence that milk exosomes are crucial for maturation and intestinal function (Gao H. N. et al., 2019; Gao R. et al., 2019; Hock et al., 2017; Li et al., 2019; Miyake et al., 2020; Reif et al., 2019; Xie et al., 2020). Strong evidence indicates that human, porcine and bovine milk exosomes aid intestinal cell growth in mice (Gao H. N. et al., 2019; Hock et al., 2017; Reif et al., 2019). Milk exosomes can also attenuate LPS-induced apoptosis (Xie et al., 2019), and prevent intestinal endothelial cell damage (R. Gao et al., 2019b; Miyake et al., 2020). They also enhance goblet cell numbers and mucin production (Li et al., 2019). They can also modify bacterial growth, and promote intestinal microbiota (Doare et al., 2018; Zhou et al., 2019). Interestingly, pasteurized fresh milk contains more bioactive milk exosomes as compared to fermented milk products like yoghurt (Yu et al., 2017). Figure 3 describes the role of microbiome derived OMVs and dietary exosomes in modulating intestinal inflammation.

### Role of Microbiota Derived Outer Membrane Vesicles in Inflammatory Bowel Disease

All biological life forms are capable of producing EVs. These include micro-organisms encountered in the intestinal tract like protozoa, archaea, bacteria and fungi (Brown et al., 2015). It has been reported that commensal microbes can modulate IECs and immune cells in the intestine by affecting maturation and other related functions. As alterations in intestinal microbiota affect the pathogenesis and development of IBD, the EVs secreted by the commensal microbes may help maintain gut immune homeostasis (Geveres et al., 2014). The involvement of extracellular factors from microbes in modulating immune response has been observed as early as 1967 (S. Chatterjee and Das, 1967). In 1967, Chatterjee and Das showed that the bacteria, *Neisseria meningitides* secreted endotoxins as cell wall blebs *in-vivo* (S. Chatterjee and Das, 1967). However, the mechanism has not yet been understood thoroughly.

Pathogenic bacteria exist in the intestines and their EVs can negatively influence gut homeostasis. They breach the intestinal barrier and facilitate mucosal invasion leading to IBD pathogenesis. *Helicobacter pylori* infection in the gut leads to gastritis, ulceration and malignancy. In 2003, a study found that it was not necessary for the bacterium to associate with the epithelial cell to cause disease, the OMVs released by the bacteria could interact with the host epithelial cell and bring about an adverse reaction (Ismail et al., 2003). Enterohemorrhagic *Escherichia coli* (EHEC) O157 EVs promote IL-8 secretion in IECs through TLR5 and TLR4/MD-2 pathway (Bielaszewska et al., 2018). They also induce apoptosis by delivering hemolysin from EHEC to endothelial cells and mitochondria (Bielaszewska et al., 2013). UGT1A1 is an important part of intestinal epithelial barrier. Fecal OMVs isolated from colitis induced rats (DSS) reduced the expression of UGT1A1 in human Caco-2 cells while, OMVs from healthy rats upregulated UGT1A1 (X. J. Gao et al., 2018). This shows that gut microbiota can regulate intestinal UGT1A1 expression. *Vibrio cholera* secretes two proteases, hemagglutinin protease (HAP) and calcium-dependent trypsin-like serine protease (VesC) in association with OMVs, these are transported to human IECs in an active form. Studies show that HAP induces apoptosis and VesC induces necrosis and increases IL-8 secretion in host cells. Data also suggests that VesC promotes intestinal colonization of *V. cholerae* in mice (Mondal et al., 2016). Also, IECs take up these EVs and stimulate the MAPK and NF-κB pathways in a nucleotide-binding oligomerization domain-containing 1 (NOD1)-dependent manner. This causes a change in the expression of IL-8, GM-CSF, CCL2, CCL20, and thymic stromal lymphopoietin (D. Chatterjee and Chaudhuri, 2013). Epithelial cadherin is essential for maintaining epithelial integrity, EVs from *Campylobacter jejuni* cleaved epithelial cadherin and facilitated the infiltration of pathogenic bacteria into IECs (Elmi et al., 2016; Boehm et al., 2018). Evidence suggests that immune responses mounted against commensal like *Bacteroides thetaiotaomicron* could trigger colitis in genetically vulnerable hosts (Hickey et al., 2015).

Microbiome derived EVs present in the host can also help maintain gut immune homeostasis. The innate immune defense of the gut relies heavily on the integrity of the epithelial layer which acts as a barrier. Apart from IECs that reinforce this barrier, probiotic bacteria can also influence barrier integrity by regulating the pro-inflammatory signals, reinforcing tight junctions between IECs and by facilitating cross-talk between commensals, gut epithelia and the mucosal immunity. *Escherichia coli* C25, is a commensal organism present in the human gut, the OMVs released by this bacterium elicits a moderate pro-inflammatory response via secretion of IL-8 *in vitro* (Patten et al., 2017). This response is beneficial to the host as healthy human intestine has a low-level inflammatory environment. OMVs from *E. coli* C25 have also shown to regulate the translocation of parent bacteria across the intestinal epithelium. This aids in maintaining a favorable symbiotic relationship with the host (Patten et al., 2017). Another probiotic bacteria, *E. coli* Nissle 1917 (EcN) helps in maintaining tight junctions by secreting OMVs and soluble factors which induce ZO-1 and claudin-14 expression, and inhibit claudin-2 expression (Alvarez et al., 2016). The pro-inflammatory effect of *E. coli* OMVs can be ameliorated by *Akkermansia muciniphila* EVs. Also, *A. muciniphila* EVs are depleted in colitis induced mice as compared to healthy mice, indicating that they play a protective role in IBD. Indeed, IBD indicators like loss of body weight, length of colon, infiltration of colon wall are maintained when *A. muciniphila* EVs are delivered orally (Kang et al., 2013). *B. fragilis* OMVs containing PSA (capsular polysaccharide) can protect against inflammation in TNBS colitis mice model. This is achieved by production of anti-inflammatory cytokines by DCs, enhancing the Treg response (Shen et al., 2012). *Pseudomonas aeruginosa* releases EVs which contains shortRNAs (sRNA), this sRNA was shown to reduce IL-8 secretion from LPS (lipopolysaccharide) induced IECs. This sRNA termed as sRNA52320 can also attenuate cytokine secretion and neutrophil infiltration (Koeppen et al., 2016). *B. thetaiotaomicron* produces a phosphatase, BTMinpp which is packaged inside OMVs, thus protecting its catalytic activity. This enzyme promotes intracellular Ca^2+^ signaling in IECs and thereby the physiological responses of the digestive system are sustained (Stentz et al., 2014). Additionally, *E. coli* OMVs (probiotic and commensal strains) induce more anti-inflammatory cytokines in explanted colonic tissue, although a modest expression of proinflammatory cytokines, such as IL-6 and IL-8 is also observed (Fábrega et al., 2016).

The host can also combat EVs from pathogenic microbes. OMVs secreted from bacteria contain cargo and this cargo is recognized by pattern recognition receptors (PRR) of the host immune system which mount an inflammatory response. The OMV released from *E. coli* contains peptidoglycan which is recognized by NOD1 receptor. This triggers the NOD1 signaling cascade, inducing the expression of pro-inflammatory molecules like NF-κB, IL-6, and IL-8 (Cañas et al., 2018). However, the OMVs may escape immune surveillance and enter IECs via clathrin-dependent endocytosis resulting in host cell DNA damage (Cañas et al., 2016). It’s not just the EVs from bacteria that regulate host cells but EVs present in the host’s physiological fluid can also interact with bacteria and promote bacterial aggregation (van Bergenhenegouwen et al., 2014). Pathogenic bacteria and commensals derived EVs regulate inter-species communication in the gut which leads to immunoregulation of signaling pathways. The source and functions of different OMV components and their function in IBD is listed in Table 2.

**TABLE 2:** Source and function of different bacterial outer membrane vesicle components in IBD.

Component	Source	Role	References
BTMinpp	*Bacteroides thetaiotaomicron*	Promotes intracellular CA^++^ signaling in IECs	Stentz et al. (2014)
Calcium-dependent trypsin-like serine protease (VesC)	*Vibrio cholera*	Plays a role in intestinal colonization of bacteria	Mondal et al. (2016)
		Induces necrosis	
		Increase IL-8 secretion in host cells	
Capsular polysaccharide (PSA)	*Bacteroides fragilis*	Induces anti-inflammatory cytokine production in DCs	Shen et al. (2012)
		Enhances Treg response	
Hemagglutinin protease (HAP)	*Vibrio cholera*	Induces apoptosis in intestinal epithelial cells	Mondal et al. (2016)
Hemolysin	Enterohemorrhagic *E.coli*	Apoptosis of endothelial cells	Bielaszewska et al. (2013)
Peptidoglycan	*Escherichia coli*	Triggers NOD1 signaling cascade	Cañas et al. (2018)
		Induces expression of IL-6, IL-8 and NF-κB	
sRNA52320	*Pseudomonas aeruginosa*	Reduces IL-8 secretion from LPS induced IECs	Koeppen et al. (2016)
		Attenuates neutrophil infiltration	

## Exosomal Components as Biomarkers for Inflammatory Bowel Disease

Micro-environmental changes take place at the site of intestinal mucosal inflammation: nutrient shortage, dysregulated oxygen supply in tissue and production of reactive nitrogen and oxygen species. Mucosal barrier, cell fate, immune regulation, and gut microbiota are affected by the consequent oxidative and hypoxia derived stress (Taylor and Colgan, 2007; Cao, 2018). An inflammatory cascade is triggered within this microenvironment through intricate crosstalk between different cell types (Marafini et al., 2019). Inflammatory cells such as granulocytes, macrophages and monocytes, and also cells of the adaptive immune system like B and T cells are sequestered within the site of active inflammation, by cytokines like IL-8 (Marafini et al., 2019). When the intestinal barrier is intact, it is very difficult for luminal antigens to approach the lamina propria. When immune cells are within the lamina propria, a tolerance system is set in place which limits induction of an immune response. On the other hand, when barrier integrity is compromised, antigens trespass the barrier and the tolerance system is invalidated. This culminates in secretion of chemokines, recruitment and infiltration of immune cells, further aggravating inflammation (Neurath, 2019). The current diagnosis of IBD involves invasive procedures like endoscopy and colonoscopy. They are also expensive, time-consuming and inconvenient for the patients. There is a need to develop a less-invasive, faster, reliable detection method for IBD.

The origin of exosomes present in the intestinal tract can be host cells, diet or microbial organisms (Chang et al., 2020). The EV content in bile samples of individuals suffering from malignant common bile stenosis was significantly higher than those seen in the bile samples of healthy controls, suggesting that the nature of EVs differs with respect to health condition of the body (Severino et al., 2017). ANXA1 is crucial for maintaining the intestinal barrier during inflammatory response; it is found in exosomes released from intestinal epithelial cells (IEC). Serum of IBD patients contain good number of such vesicles, making it a promising biomarker for intestinal inflammation (Leoni et al., 2015). Proteasome Subunit Alpha type 7 (PSMA7) is found in oral exosomes secreted by the oral mucosal cells of IBD patients. The expression of PSMA7 isolated from saliva in IBD patients is remarkably different than those in healthy controls and is a good biomarker candidate (Zheng et al., 2017). More exosomal characterization studies on biological fluids from IBD patients would help in identifying novel biomarkers that could help in early diagnosis of IBD.

## Effect of Exosomes on Current Inflammatory Bowel Disease Treatment

IBD treatment involves anti-inflammatory drugs, immune system suppressors, and biologics supplemented with antibiotics and dietary supplements. Biologics are the newest category of drugs in IBD treatment. Most of them are monoclonal antibodies which neutralize inflammation causing proteins in the body. The most common form of biologics administered to IBD patients are TNF blocking agents e.g.,: Infliximab, Adalimumab, Golimumab, and Certolizumab. Other biologics used in IBD treatment are Vedolizumab, which is a α_4_β_7_ blocker and Ustekinumab, which blocks the IL12-IL23 pathway.

A recent study detected α_4_β_7_ integrin expression on the surface of circulating exosomes from serum of IBD patients being treated with Vedolizumab (VDZ) (Domenis et al., 2020). These α_4_β_7_ expressing exosomes were able to bind to VDZ competitively *in-vitro*. In IBD treatment, VDZ acts by binding to the α_4_β_7_ integrin found on a subset of T cells and prevents their trafficking into the gut. This data suggests that exosomes could be involved in mediating VDZ resistance in IBD patients by sequestration of therapeutic molecules into vesicles. Most IBD patients on treatment with a biologic drug respond poorly to a second biologic drug (Gisbert ad Chaparro, 2020). The study by Domenis et al. (2020) reports that the serum exosomal concentration and vesicle surface expression of α_4_β_7_ integrin was greater in the serum of patients on TNF blocking drugs compared to TNF naïve patients. The resultant exosomal sequestration of VDZ also increased in patients on TNF blocking drugs suggesting that previous treatment with a biologic drug alters the expression of α4β7 integrin and binding capacity of circulating exosomes. This potentially explains the reduced efficacy of VDZ in IBD patients who were previously treated with TNF blocking agents.

To date this is the only study correlating the effect of exosomes on IBD treatment modalities. Further studies in this direction would help design better treatment strategies that could increase drug bioavailability.

## Role of Exosomes in Inflammatory Bowel Disease Therapy

Exosomes are natural carriers of functional RNAs, proteins and have the characteristics to be employed for transporting drugs or biologicals. They can also be employed for tissue repair and regeneration. Modification of exosomes either biologically or chemically will boost their therapeutic potential (Ocansey et al., 2020). IBD is characterized by disruption of the immune system and abnormal stimulation of immune cells. As mentioned in this review, DC derived EVs through immune excitation or repression mitigate IBD progression. Hence, it is plausible that immune cells derived EVs may be the new therapeutic intervention for IBD.

The interest in EV research also propels the exploration of artificial nanoparticles as tool for disease treatment. Studies have looked at EV-like nanoparticles as treatment for IBD. Bioadhesive chitosan has been developed, which can be delivered by oral route to accumulate in a safe and specific way to affected areas in the GI tract (Han et al., 2019). Intestinal organoids containing 5-ASA-loaded nanoparticles have been used to relieve IBD (Davoudi et al., 2018). Mannosylated bioreducible cationic polymers have been used to synthesize RNAi (RNA interference) nanoparticles to lower cell-death and improve treatment efficacy in IBD (Xiao et al., 2013). EVs are bio-compatible and stable than artificial nanoparticles as they are innate in nature, being derived from cells and microbes. EVs can also be engineered, enhancing their role in treatment and therapy in IBD (Hood and Wickline, 2012; Hood, 2016). Promising results have been observed with Rifaximin loaded EVs (Kumar and Newton, 2017; García-Manrique et al., 2018). These studies demonstrate that EVs have a potential use in drug delivery.

Breast milk is known to have immunoglobulins, approximately 15 years ago it was discovered that it contains immune-modulatory EVs (Admyre et al., 2007). Since then milk EVs have received considerable attention and a lot of studies have been conducted to understand the process of drug delivery in the intestinal tract. EVs are resilient to gastric/pancreatic digestion (Hata et al., 2010), indicating that breast milk EVs are delivered to intestines of infants (Kahn et al., 2018). Milk derived EVs were also observed in human intestinal crypt-like cells, indicating a possible role in neonatal mucosal immunity (Liao et al., 2017). Several studies recommend that IEC viability, stem-cell activity and proliferation capacity increase when treated with milk derived EVs (T. Chen et al., 2016; Gao H. N. et al., 2019; Hock et al., 2017). Breast milk EVs can prevent cell death in IECs and decrease necrotizing enterocolitis (NEC) (Lucas and Cole, 1990; Martin et al., 2018). They also offer the possibility of delivering drugs in milk (Schanler et al., 1992; Zamrik et al., 2018). Small molecules such as siRNA are ideal candidate for therapy but they are unstable during delivery. A 2017 study showed that AF488 could be encapsulated in milk whey and EVs would ensure their delivery to Caco-2 cells (Shandilya et al., 2017).

Protein cargo content of EVs have also been researched. Intestinal EVs contain high levels of the cytokine, TGF-β1 which induces Treg cells and immunosuppressive DCs in IBD mice (Jiang et al., 2016). Meanwhile, ANXA1 containing exosomes stimulates epithelial cell repair in a colitis mice-model. Increased amount of ANXA1 is also observed in sera of IBD patients, the increase is directly proportionate to disease severity (Leoni et al., 2015). This provides strong evidence to use EV components as biomarkers for disease severity and IBD progression but may also have a role in therapy. Anti-tumor immunity can be induced by mature DC-derived exosomes carrying tumor antigens in *in-vitro* trials (Yang et al., 2010). Most translational EV studies are still in the pre-clinical stages with data available from animal and cellular models. Additional research would help in exploring the application of EVs in diagnostics and therapeutics in a clinical setting.

## Discussion

Evidence from recent studies on exosomes in particular and EVs in general indicate that these extracellular vesicles are capable of modulation of genes and cellular function and can thereby regulate inflammation and immune response. Based on our review of published studies, it is evident that exosomal cargo is strongly associated with the nature of the cells which produces them. Exosomal components are optimal for use as biomarkers as they can be extracted with minimal invasive techniques from most biological fluids like blood, urine, saliva, and feces. Existing data in IBD is limited to initial characterizations of exosomes in blood and fecal samples. Further studies are required to identify sensitive and specific markers of mucosal inflammation, and ideally inflammatory pathways. This could guide treatment decisions when several mechanisms of drug action are available to clinicians. As researchers we need to make efforts to develop robust methodologies for isolation of exosomes from fecal samples to ensure results are reliable and reproducible.
